# Natural Bioactive Products with Antioxidant Properties Useful in Neurodegenerative Diseases 2020

**DOI:** 10.1155/2021/6262316

**Published:** 2021-02-10

**Authors:** Francisco Jaime Bezerra Mendonça-Junior, Marcus Tullius Scotti, Eugene N. Muratov, Luciana Scotti, Anuraj Nayarisseri

**Affiliations:** ^1^Postgraduate Program in Natural and Synthetic Bioactive Products, Federal University of Paraiba, João Pessoa, PB, Brazil; ^2^Laboratory of Synthesis and Drug Delivery, Department of Biological Science, State University of Paraiba, João Pessoa, PB, Brazil; ^3^Laboratory for Molecular Modeling, UNC Eshelman School of Pharmacy, University of North Carolina, Chapel Hill, North Carolina 27599, USA; ^4^Teaching and Research Management-University Hospital, Federal University of Paraiba, João Pessoa, PB, Brazil; ^5^In Silico Research Laboratory, Eminent Biosciences, Indore, 452010 Madhya Pradesh, India; ^6^Bioinformatics Research Laboratory, LeGene Biosciences Pvt. Ltd., Indore, 452010 Madhya Pradesh, India

Neurodegenerative diseases (NDs) are a group of diseases that affect millions of people worldwide and which are characterized by the progressive degeneration of the nervous system, compromising cognitive and/or motor functions. The most common NDs are Parkinson's disease (PD), Alzheimer's disease (AD), Huntington's disease, Prion disease, amyotrophic lateral sclerosis (ALS), and multiple sclerosis (MS). These pathologies have multifactorial causes, many of which are not yet fully understood; however, recent studies show that ageing and oxidative stress play important roles in the appearance and evolution of these pathologies; to which, the therapeutic options available are not curative and only slow their progression. Despite this, it is very well established that antioxidant therapy that can control free radicals and reactive oxygen species (ROS) levels is a promising strategy to delay, prevent, and/or treat NDs.

In this context, natural bioactive compounds isolated from plants, animals, bacteria, fungi, and algae are widely known and employed since the beginning of human life on Earth, to treat many pathologies. In recent years, in silico, in vitro, and in vivo studies, performed with natural products and its isolated bioactive compounds, have proven its biological activities and large therapeutic benefits, including its antioxidant properties, reducing ROS levels, and modulating the cellular redox balance, thus stating its great potential to delay, prevent, and/or treat NDs.

In this special issue, articles were selected that address new therapeutic alternatives on the antioxidant role with related neuroprotective effects of natural bioactive compounds in the prevention/treatment or improvement of neurodegenerative diseases. This special issue compiles eighteen (18) manuscripts including three (3) reviews and fifteen (15) research papers, which show recent research about the discovery of plant-derived antioxidants with application in NDs.

In their minireview, R. Chandran and H. Abrahamse describe several plants, plant parts, and isolated phytochemicals (especially phenolic compounds) with the potential to prevent the progression of neurodegenerative diseases due to its antioxidant properties. In the second review, A. G. Miranda-Díaz, E. G. García-Sanchez, and A. Cardona-Muñoz report the pro-oxidant effect of consumption of processed meat and the neuroprotective effects of some foods constituents with antioxidant properties, like melatonin, *N*-acetylcysteine, vitamin B3, ascorbic acid, and vitamin D, on brain health in PD. In the review by J. M. Silva et al., the authors summarize the natural bioactive compounds with antioxidant properties useful against ALS.

Fourteen of the fifteen research articles addressed to deal with the proof of the neuroprotective activity associated with the antioxidant properties of plants, mushrooms, plant extracts, isolated phytoconstituents, herbal medicines, and compositions containing phytochemicals. The only article that is out of this theme is the work of M. S. Maia et al. which performed a virtual screening based on studies of QSAR (quantitative structure-activity relationship), molecular docking, and ADMET (absorption, distribution, metabolism, excretion, and toxicity) properties, allowing to identify lignans with potential multitarget action against enzymes related to the oxidative pathway which represent a potential alternative for AD treatment.

K. Lei et al. demonstrated that the flavonoid baicalin improved PD model's behavioral performance, reducing dopaminergic neuron loss in the substantia nigra. Additionally, baicalin has the ability to protect dopaminergic neurons against ROS, decreasing the expression of the transcription factors such as *α*-synuclein and CCAAT/enhancer-binding protein *β* (C/EBP*β*). Y. Zhang et al., using a model of age-related macular degeneration (AMD), demonstrated that solid dispersion of the flavonoid apigenin is able to reduce retinal oxidative injury by upregulation of autophagy and of the expression of antioxidant enzymes through the Nrf2 pathway. Q. Chu et al. demonstrated that purified flavones (3-caffeoylquinic acid, 5-caffeoylquinic acid, quercetin-3-O-rutinoside, and kaempferol-3-O-rutinoside) from the vine part of *Tetrastigma hemsleyanum* present in vitro and in vivo neuroprotective effects through mitogen-activated protein kinase (MAPK) pathways in models induced by glutamic acid. Q. Zhu et al. found that the dihydro flavonoid naringin is capable of increasing tolerance to oxidative stress and delaying the evolution of ageing-related diseases like AD and PK, via the transcription factor DAF-16.

I. Rjeibia, et al. demonstrated that polysaccharides from the pulps of *Crataegus azarolus L.* var. *Aronia* exhibit the neuroprotective activity mediated by its antioxidant, *α*-amylase, and acetylcholinesterase inhibitory activities. S. S. Singh et al.'s group observed that chlorogenic acid has a neuroprotective effect and decreased the loss of dopaminergic neurons in the PD mouse model. The effect was associated with the attenuation of mitochondrial dysfunction, inhibition of proapoptotic proteins caspase-3 and Bax, and with phosphorylation of GSK3*β* via activating Akt/ERK signaling in the mitochondrial intrinsic apoptotic pathway. R.-L. Li et al. demonstrated that hydroxy-*α*-sanshool (isolated from *Zanthoxylum bungeanum* pericarps) promotes neuroprotection by reduction of intracellular ROS and suppression of oxidative stress caused by H_2_O_2_.

D. Liao et al. observed that the polyphenol curcumin reduces the depressive state by decreasing the expression of oxidative stress markers and activating the Nrf2-ARE pathway. D. Disana et al. demonstrated that the secoiridoid glycoside amarogentin (isolated from *Gentiana rigescens* Franch) promotes anti-ageing and neuroprotective effects by reduction of oxidative stress, promoted by the increase of catalase, superoxide dismutase, and glutathione peroxidase activities. A. Litwiniuk et al. demonstrated that alpha-linolenic acid stimulates the release of insulin from astrocytes, and these astrocytes were capable to protect cells against A*β*1-42-induced mitochondrial dysfunction promoting neuroprotection.

J. Giacomettil and T. Grubić-Kezele demonstrated that olive leaf polyphenols promote neuroprotection through the reduction of oxidative stress, regulation of microglia and SIRT1, and maintaining the myelin sheath integrity, which can be effective for the treatment of multiple sclerosis and other neurodegenerative diseases. A. Agapouda et al. evidenced the anti-ageing and neuroprotective effects of Honeybush extracts (Cyclopia spp.). The authors associated these properties to the extract's ability to recover the mitochondrial function under oxidative stress conditions.

N. S. El Sayed and M. H. Ghoneum proved that Antia, a natural product extracted from yamabushitake mushroom, can exert protective effects attenuating cognitive dysfunction against sporadic AD. The action is associated to its interference in amyloidogenic, inflammatory, and oxidative stress pathways, including the JAK2/STAT3 pathway. M. Yi et al. investigated the effects on the cognitive performance and the interference of Bushen Tiansui formula on the metabolomic and lipidomic profiles in the cerebral cortices of the rat AD model. The authors found that the Bushen Tiansui formula can restore the metabolic balance acting on the metabolism of sphingolipids, glycerol phospholipids, alanine, aspartate, glutamine, and glutamate, thus exerting its neuroprotective effect.

Taken together, these studies provide readers with an updated view of the evidence and mechanisms of action of herbal medicines, compositions containing natural products, and several classes of secondary metabolites including polysaccharides, flavonoids, lignans, polyphenols, iridoids, fatty acids, phenolic acids, and alkyl amides in reducing oxidative stress in the context of NDs. We are sure that these findings will be useful and will contribute to the success of new therapies for NDs.

